# Detecting non-content-based response styles in survey data: An application of mixture factor analysis

**DOI:** 10.3758/s13428-023-02308-w

**Published:** 2023-12-21

**Authors:** Víctor B. Arias, Fernando P. Ponce, Luis E. Garrido, María Dolores Nieto-Cañaveras, Agustín Martínez-Molina, Benito Arias

**Affiliations:** 1 Department of Personality, Assessment and Psychological treatment, Faculty of Psychology, University of Salamanca, Av. De la Merced, 109 Salamanca, Spain; 2 Faculty of Psychology, University of Talca, Talca, Chile; 3 Pontificia Universidad Católica Madre y Maestra, Santiago de los Caballeros, Dominican Republic; 4 Universidad Antonio de Nebrija, Madrid, Spain; 5 Autonomous University of Madrid, Madrid, Spain; 6 University of Valladolid, Valladolid, Spain

**Keywords:** Non-content-based, Responding, Careless responding, Insufficient-effort responding, Data cleaning, Factor mixture analysis

## Abstract

It is common for some participants in self-report surveys to be careless, inattentive, or lacking in effort. Data quality can be severely compromised by responses that are not based on item content (non-content-based [nCB] responses), leading to strong biases in the results of data analysis and misinterpretation of individual scores. In this study, we propose a specification of factor mixture analysis (FMA) to detect nCB responses. We investigated the usefulness and effectiveness of the FMA model in detecting nCB responses using both simulated data (Study 1) and real data (Study 2). In the first study, FMA showed reasonably robust sensitivity (.60 to .86) and excellent specificity (.96 to .99) on mixed-worded scales, suggesting that FMA had superior properties as a screening tool under different sample conditions. However, FMA performance was poor on scales composed of only positive items because of the difficulty in distinguishing acquiescent patterns from valid responses representing high levels of the trait. In Study 2 (real data), FMA detected a minority of cases (6.5%) with highly anomalous response patterns. Removing these cases resulted in a large increase in the fit of the unidimensional model and a substantial reduction in spurious multidimensionality.

**Supplementary Information:**

The online version contains supplementary material available at 10.3758/s13428-023-02308-w.

## Introduction

In self-report measures, some respondents pay insufficient attention or do not make enough effort, resulting in responses with validity issues (Arias et al., 2020a; Curran, 2016; Dunn et al., 2018; Huang et al., 2015). This phenomenon has been referred to as random responding, content-independent responding, inconsistent responding, careless responding, or insufficient-effort responding, among other names (Johnson, 2005; Hong et al., 2020; Huang et al., 2012; Meade & Craig, 2012; Nichols et al., 1989). Several causes of misresponding have been identified, such as difficulty understanding an item’s content, carelessness and inattention, poor processing due to demotivation or fatigue, use of response heuristics, difficulty verifying the semantic polarity of an item, and automated bot responses to online surveys with monetary rewards (Baumgartner et al., 2018; Curran, 2016; DeSimone & Harms, 2018; Dupuis et al., 2018; Johnson, 2005; Swain et al., 2008; Weijters et al., 2013).

In this study, we used the general term non-content-based (nCB) responses to refer to responses that, for a variety of reasons, completely or partially ignore the meaning and semantic direction of the items and therefore do not validly represent the trait or state being measured. nCB responses are usually presented in the form of response styles with varying degrees of systematicity (Arias et al., 2020b; DeSimone & Harms, 2018; Huang et al., 2012). (Dis)acquiescent and middle response styles tend to concentrate responses in a limited scale range, ignoring the meaning and polarity of items. The random style is characterized by the use of the full range of response categories, possibly in an attempt to simulate a thoughtful response. These styles can vary in severity to the extent that they affect all or part of the response vector (Hong et al., 2020).

The detrimental effects of nCB data have been well documented: increased risk of type I error in decision-making between competing models, replication problems between studies with different proportions of nCB responses, spurious relationships between truly unrelated variables, artificial deflation or inflation of the internal consistency of data, appearance of factors other than those theoretically expected, obscured effects of experimental manipulation, and severe perturbations in the factorial structure of data (Arias et al., 2020a; Curran, 2012; García-Batista et al., 2021; Goldammer et al., 2020; Huang et al., 2015; Johnson, 2005; Kam & Meyer, 2015; Maniaci & Rogge, 2014; Steinmann et al., 2022; Wood et al., 2017; Woods, 2006).

One of the common consequences of nCB data is the appearance of spurious wording/method factors due to inconsistent responses to positive and reverse-keyed items (Ponce et al., 2023). Various approaches have been proposed to model this inconsistency, usually by specifying additional factors (DiStefano & Motl, 2006; Eid, 2000; Gnambs et al., 2018; Horan et al., 2003; Marsh et al., 2010; Michaelides et al., 2016; Savalei & Falk, 2014; Tomás & Oliver, 1999; Weijters et al., 2013). However, recent studies using mixture models suggest that the phenomenon represented by the wording/method factor is not generalizable to the whole sample: on the contrary, a large proportion of spurious variance is due to a limited proportion of response vectors (Arias et al., 2020a; García-Batista et al., 2021; Ponce et al., 2021; Reise et al., 2016; Steinmann et al., 2021, 2022; Yang et al., 2018). Therefore, although modeling the wording variance helps to reveal the true structure of data, the estimates of the trait in the contaminated vectors remain biased, which may affect important properties of the data, such as the accuracy of the estimators, validity coefficients, or measurement invariance (Arias et al., 2020a; Nieto et al., 2021; Tomás et al., 2015).

If the trait estimates in the individual nCB responses were biased, it would be logical to screen and consider eliminating them from the database, especially when analyzing individual scores. Over the past few decades, several approaches have been developed to detect nCB responses. Many screening techniques attempt to detect nCB vectors through post hoc statistical means, such as the Mahalanobis distance, even–odd correlation, personal reliability, inter-item standard deviation, or various indices of personal fit based on parametric item response theory models (Curran, 2012; Emons, 2008; Ferrando, 2015; Hong et al., 2020; Karabatsos, 2003; Schneider et al., 2018; Zijlstra et al., 2007). A few studies evaluate the diagnostic capacity of screening methods (Conijn et al., 2019; Goldammer et al., 2020; Hong et al., 2020; Huang et al., 2012; Meade & Craig, 2012; Niessen et al., 2016). A common finding in these studies is that no single method can satisfactorily detect all possible nCB response styles; hence, these studies recommend combining multiple methods (Hong et al., 2020; Meade & Craig, 2012).

Factor mixture analysis (FMA; Lubke & Muthén, 2005) has not been widely used to detect anomalous responses although the model has potential utility in the study of nCB response styles. FMA combines the common factor model with latent class analysis, involving a categorical latent variable (class) and one or more continuous latent variables (factors). Recently, FMA applications have been developed to identify individuals who respond inconsistently to positive and reverse-keyed items due to inattention and carelessness or difficulties in reading comprehension (Arias et al., 2020a; Kam & Fan, 2020; Steinmann et al., 2021; Ulitzsch et al., 2022).

In this study, we propose an FMA model for detecting responses that are not based on item content and that manifest themselves as generalized response styles. The FMA model presented here can be considered confirmatory since it includes a priori theoretical constraints aimed at identifying two non-invariant and qualitatively different classes: thoughtful responses and responses not based on item content. The following sections explain the underlying logic of the model.

## Factor mixture model

A factor mixture model (Arminger et al., 1999; Dolan & Van der Maas, 1998; Lubke & Muthén, 2005, 2007; Yung, 1997) is a hybrid model that combines latent class analysis (LCA) and factor analysis (FA) and can be understood as a latent class model in which each class has its own common factor. In the basic LCA model, classes are categorical variables in which intraclass variability is not allowed; that is, individual differences are fully explained by the class (Hagenaars & McCutcheon, 2002). However, this assumption can sometimes be too restrictive (Lubke & Muthén, 2007). To alleviate this restriction, FMA allows for individual intraclass differences by estimating a factor model for each class. Moreover, FMA can determine the degree of similarity between the parameters of the factor models, ranging from configural to strict invariance. Consequently, FMA can function as a multigroup factor model in which the groups are unknown a priori (Clark et al., 2013).

## Specification of a mixture factor model for detecting nCB responses

Our starting hypothesis is that any survey dataset can be a mixture drawn from two qualitatively different populations: (a) individuals who have paid attention to and understood the meaning of questions so that their responses are expected to be related to the content of items, and (b) individuals who have not paid sufficient attention to or understood what they were asked to do so that the relationship between their responses and the content of items is null or severely biased. The two groups can be represented as non-invariant latent classes, which we refer to as the “non-content-based” (nCB) and the “content-based” (CB) classes. The nCB class comprises participants who employed response styles on the whole or most of the scale. On the other hand, the CB class consists of individuals who responded reflexively according to item’s meaning. In the following paragraphs, we present the rationale for the model and its specification.

In a standard factor model, the response *y* of a subject *j* to an item *i* can be defined as

$${y}_{ij}={\mu}_i+{\lambda}_i{\eta}_j+{e}_{ij}$$

where *μ*_*i*_ is the intercept of item *i*, *λ*_*i*_ is the vector of factor loadings of item *i*, *η*_*j*_ is the vector of common factors of subject *j*, and *e*_*ij*_ is the error term of subject *j* in item *i*. The subscript *j* implies that the parameter varies across individuals; therefore, *λ*_*i*_ and *μ*_*i*_ are considered fixed coefficients that are invariant across individuals but may vary across items. Because *μ*_*i*_ is the expected score on an item for an intermediate level of a trait, *μ*_*i*_ can be interpreted as an indicator of the “intensity” of the item, signifying the degree of latent trait associated with the probability of obtaining a particular score, analogous to how thresholds are interpreted in item response theory models. The parameter *λ*_*i*_ is the slope of an item, which is the rate at which the expected score varies as a function of an individual’s level of the trait. Thus, *λ*_*i*_can be understood as the discriminative ability of the item along the latent continuum.

In general, the goal of an instrument is to include items with appropriate discrimination (λ) and varying intensities (μ) to provide information across a sufficiently broad trait range. Consequently, the estimates of loadings and intercepts depend essentially on the interaction between (a) the meaning and semantic polarity of an item and (b) the trait distribution in each particular sample.

What would happen if respondents did not pay attention to or understand the content of the items? If the respondent pays attention and understands the content of the items, the items act as stimuli that elicit responses that measure an underlying latent variable. As noted above, these stimuli differ in intensity (intercepts) and the degree to which they discriminate between regions of the latent variable (loadings). However, if items are not attended to or correctly processed, they lose their properties as stimuli because their relationship to the latent variable depends on item meaning. Consequently, the item response ceases to represent the individual’s position on the trait of interest and instead represents something qualitatively different: an extreme form of idiosyncratic use of the response scale, expressed as nCB response styles (e.g., random, dis-acquiescent, patterned, or middle responding).

It has been noted that the estimates of μ and λ have the same value for all individuals but may vary across items. Moreover, the value of μ and λ largely depends on the meaning of each item. Applying these considerations to the case of nCB data, we propose the following hypotheses regarding the model parameters and the structure of nCB data:

First, since a response style is unrelated to the item’s content and acts consistently across items, there can be no variance in item intensity. Thus, in a dataset of nCB responses, the intercept for Item 1 will be identical to that for every other item (i.e., *μ*_1_ = *μ*_2_ = *μ*_*n*_) because, for an inattentive respondent, all items have no meaning. Similarly, differences in the magnitude and sign of factor loadings are not expected because (a) the discriminative power of items essentially depends on the interaction between the meaning of an item (which is ignored by the respondent) and the individual’s level on a trait (which is not measured) and (b) the sign of the factor loadings depends on the semantic of the item (which is ignored by the respondent). Thus, for a set of nCB responses, *λ*_1_= *λ*_2_= *λ*_*n*_.

Second, nCB responses are expressed as response styles (e.g., acquiescent and random). Such styles can be depicted as discrete latent classes because latent classes are internally homogeneous but heterogeneous with respect to each other (Borsboom et al., 2016). However, taken together, the responses from different nCB styles may result in an apparently continuous distribution that can be modeled as a factor, although its true structure is essentially categorical (Arias et al., 2020b). Therefore, the factor specified in the nCB class has no substantive interpretation in terms of a continuously distributed latent variable; we consider the factor a mathematical artifact resulting from the mixture of different discrete latent classes.

**Fig. 1:**
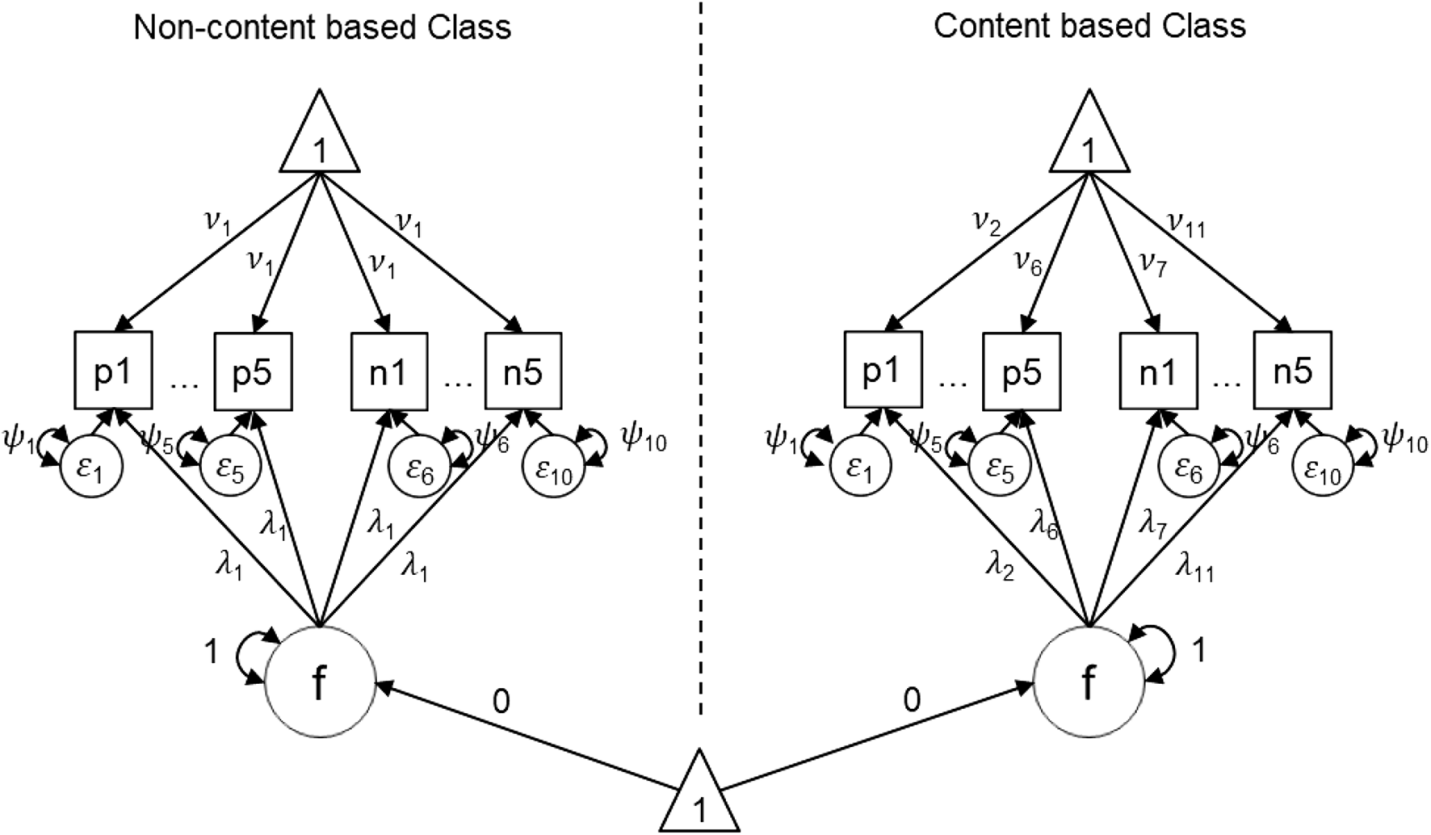
Restricted factor mixture analysis model (example with ten items, half reverse-keyed). p1–p5, positively worded items; n1–n5, negatively worded items; f, factor; 𝜓, residual; 𝜈, item intercept; 𝜆, item factor loading; Φ, factor variance (1); and 𝜶, factor mean (0)

To operationalize this hypothesis, we specify a one-dimensional FMA model with two latent classes (see Fig. 1).

- Class 1 aims to capture nCB response vectors. All intercepts (𝜈) are set equal to each other, and all loadings (𝜆) are set equal to each other, with the variance (Φ) of the factor set to 1 and its mean (𝜶) set to 0 for identification.
- Class 2 aims to capture content-based response vectors. Loadings and intercepts are estimated without constraints, with the variance (Φ) of the factor set to 1 and its mean (𝜶) set to 0. Given the equality constraints, the CB class measurement model is congeneric, while the nCB class measurement model can be considered essentially tau-equivalent.

The class-specific residuals (𝜓) are not estimated for two reasons: (a) to focus interclass differences only on loadings and intercepts and (b) to prevent the nCB class from capturing differences in the structure of the residuals related to minor violations of conditional independence not specified in the overall model. In addition, empirical underidentification is likely to occur when the size of one of the classes is small relative to the number of class-specific parameters (Lubke & Luningham, 2017). To avoid underidentification problems, homoscedasticity across classes was assumed in the residual variances of the items (Steinmann et al., 2021).

The FMA model estimates the extent to which each response vector is compatible with each factor model by assigning a value between 0 and 1, estimating for each case its posterior probability of belonging to Class 1 or 2. As a cutoff, we consider cases with 50% or greater posterior probability of belonging to Class 1 as potential nCB responses. Finally, the model must be estimated without prior recoding of the reverse-keyed items.

We conducted two studies to investigate the ability of the restricted FMA model to detect nCB responses. The first study aimed at estimating the diagnostic capacity of FMA using simulated data, while the second study investigated aspects related to the validity and usefulness of the model on real data.

## Study 1 (simulated data)

In the first study, we investigated the ability of FMA to detect nCB responses. We used simulated datasets with different prevalence of nCB cases (from 3% to 40%), representing 5 response styles (i.e., acquiescent, dis-acquiescent, middle responding, random responding, and patterned responding), with both mixed-worded and positive-only item scales.

In addition, we examined the extent to which the classification index produced by the FMA is confounded by the substantive trait. An estimator of data quality must be independent of the trait to avoid selection bias (Conijn et al., 2019; Thomas & Clifford, 2017). However, in mixed-worded scales, individuals with medium levels of the latent trait may produce inconsistent responses to positive and negative items (Kam et al., 2021; Ponce et al., 2023). To ensure that a screener does not introduce selection bias, the correlation between the screener and the trait estimates should be close to 0, the proportion of false positives should be small, and there should not be a part of the trait range in which the screener scores are systematically high.

### Instruments and participants

We used two simulated samples, which we called “CB respondents” and “nCB respondents.” The datasets and R scripts generated and/or analyzed in this study are available in the Open Science Framework repository (https://osf.io/fy59v/?view_only=580d587f6c1e4b49beae6d270ee07078). Appendix A contains an annotated example of MPlus code for estimating the FMA model.

CB responses were simulated using a Monte Carlo model (Muthén & Muthén, 2002). First, we obtained the population parameters of a one-dimensional model estimated on the matrix of polychoric correlations of responses to 14 extraversion items (with seven reverse-keyed items) from 203,090 American adults with no missing data (ages 17–76, *M* = 25, and *SD* = 10.2; 62% female). This sample was drawn from the data used by Johnson (2014) in studies on the structure and properties of IPIP-NEO-300. The one-dimensional model fitted the real data reasonably well (root mean square error of approximation [RMSEA] = 0.086, comparative fit index [CFI] = 0.958, and Tucker–Lewis index [TLI] = 0.947). Next, we simulated the responses to 14 ordinal items with five response categories, using the parameters obtained from real data (Table 1 shows the standardized parameters of the base model). Based on the parameters of the real data, we built two simulation models: one simulating responses to seven positive and seven reverse-keyed items and another one simulating responses to 14 positive items. In the case of the positive item scale, we used the same parameters, with the signs of the loadings and the values of the thresholds adjusted to the polarity of the items.

**Table 1:** Model parameters in simulations conducted in Study 1

Item	λ_i_	τ_1_	τ_2_	τ_3_	τ_4_
Item 1	0.716	– 1.605	– 0.805	– 0.372	0.668
Item 2	0.768	– 1.848	– 0.997	– 0.486	0.817
Item 3	0.584	– 2.145	– 1.418	– 0.840	0.551
Item 4	0.710	– 0.967	– 0.250	0.164	0.925
Item 5	0.716	– 1.669	– 0.921	– 0.408	0.656
Item 6	0.628	– 1.540	– 0.597	0.006	1.288
Item 7	0.571	– 1.203	– 0.639	– 0.100	0.622
Item 8	– 0.640	– 0.955	– 0.174	0.162	0.933
Item 9	– 0.704	– 1.377	– 0.442	– 0.034	0.953
Item 10	– 0.787	– 1.784	– 0.859	– 0.396	0.601
Item 11	– 0.458	– 2.153	– 1.315	– 0.883	0.334
Item 12	– 0.681	– 1.142	– 0.254	0.379	1.129
Item 13	– 0.723	– 1.220	– 0.337	0.095	1.019
Item 14	– 0.573	– 0.927	0.166	0.788	1.620

**Note**. λ_i_ = item slope; τ_i_ = item thresholds.

Second, we simulated the nCB data by emulating five response styles: acquiescent, dis-acquiescent, middle responding, random responding, and patterned responding (Arias et al., 2020b; Baumgartner & Steenkamp, 2001; Curran & Denison, 2019; DeSimone & Harms, 2018; Messick, 1991; Weijters et al., 2013). Each response style pattern was generated according to the probability of selecting each response category (Table 2).

**Table 2:** Simulated response probabilities by response style

Response style	Category 1	Category 2	Category 3	Category 4	Category 5
Acquiescent	.050	.050	.100	.400	.400
Dis-acquiescent	.400	.400	.100	.050	.050
Middle	.025	.075	.800	.075	.025
True random	.200	.200	.200	.200	.200
Patterned (odd items)	.050	.050	.200	.350	.350
Patterned (even items)	.350	.350	.200	.050	.050

To simulate the acquiescent, dis-acquiescent, and middle response styles, the highest response probabilities were assigned to the high, medium, and low categories of the response scale. To simulate the true random style, an identical probability (.20) was assigned to all categories. However, the ability of humans to produce truly random responses without prior training has been questioned (Neuringer, 1986). Consequently, we included a style called “patterned” that mimics the tendency to use the full range of a scale to fake thoughtful responses (Curran & Denison, 2019; DeSimone & Harms, 2018), but in a nonrandom manner. In contrast to the random style, responses in the patterned style are not independent of each other; rather, each response depends in part on the immediately preceding response creating “patterned” vectors (Curran & Denison, 2019). To simulate the patterned style, different response probabilities were assigned to odd and even items to mimic the tendency of this style to shift between both sides of the response scale. We generated the individual responses according to each of the five styles. In each simulation condition, the total nCB responses consisted of a balanced mixture of the five response styles. For example, in a simulation condition with 1800 CB cases and 200 nCB cases (10% prevalence), 40 nCB cases were acquiescent, 40 were dis-acquiescent, and so on.

Table 3 shows 30 simulation conditions according to the total sample size (500, 2000, and 5000), the semantic polarity of the items (a balanced scale with half of the items reversed and a scale with all items phrased in the same direction), and the proportion of nCB data (prevalence of 3%, 5%, 10%, 20%, and 40%). Each of the 30 models was estimated 100 times (3000 datasets). All analyses were conducted with MPlus 8.2 (Muthén & Muthén, 1998–2017) and R (R Core Team, 2021).

**Table 3:** Simulation conditions by item polarity and proportion of nCB data

Condition	Sample size	Item polarity	Proportion of nCB data (*n*)
A1	500	Half positive (7) and half reversed-keyed (7)	3% (15)
A2			5% (25)
A3			10% (50)
A4			20% (100)
A5			40% (200)
B1	2000	Half positive (7) and half reversed-keyed (7)	3% (60)
B2			5% (100)
B3			10% (200)
B4			20% (400)
B5			40% (800)
C1	5000	Half positive (7) and half reversed-keyed (7)	3% (150)
C2			5% (250)
C3			10% (500)
C4			20% (1000)
C5			40% (2000)
D1	500	All positive-keyed (14)	3% (15)
D2			5% (25)
D3			10% (50)
D4			20% (100)
D5			40% (200)
E1	2000	All positive-keyed (14)	3% (60)
E2			5% (100)
E3			10% (200)
E4			20% (400)
E5			40% (800)
F1	5000	All positive-keyed (14)	3% (150)
F2			5% (250)
F3			10% (500)
F4			20% (1000)
F5			40% (2000)

**Note**. nCB = Non-content based responses.

### Methods

We analyzed diagnostic accuracy using receiver operating characteristic (ROC) curves and sensitivity and specificity indices associated with the theoretical cutoff point (probability of belonging to the nCB class ≥ .50). The area under the curve (AUC) can range from .50 to 1, where 1 represents perfect diagnostic accuracy and .50 represents zero accuracy (i.e., no difference from chance). Conventionally, AUC values above .97 are considered excellent, .90 to .97 very good, .75 to .90 good, .60 to .75 fair, and .50 to .60 poor (Krzanowski & Hand, 2009). Sensitivity is the proportion of individuals with the target condition identified by a diagnostic test (i.e., true positives). Specificity is the proportion of individuals who, while not exhibiting the target condition, were not identified by the test (i.e., true negatives).

We also estimated positive predictive values and negative predictive values (PPVs and NPVs, respectively). PPV is the proportion of positive cases flagged as positive. NPV is the proportion of negative cases flagged as negative. PPV and NPV provide a detailed assessment of the usefulness of a screener. In the case of large discrepancies between PPV and NPV, a high NPV is desirable when it is important to avoid false positives (in the case of nCB responses, to avoid screening valid cases), whereas a high PPV is desirable when it is important to detect as high a proportion of true positives as possible.

### Results

#### Conditions A1–C5 (mixed-worded scale)

**Fig. 2:**
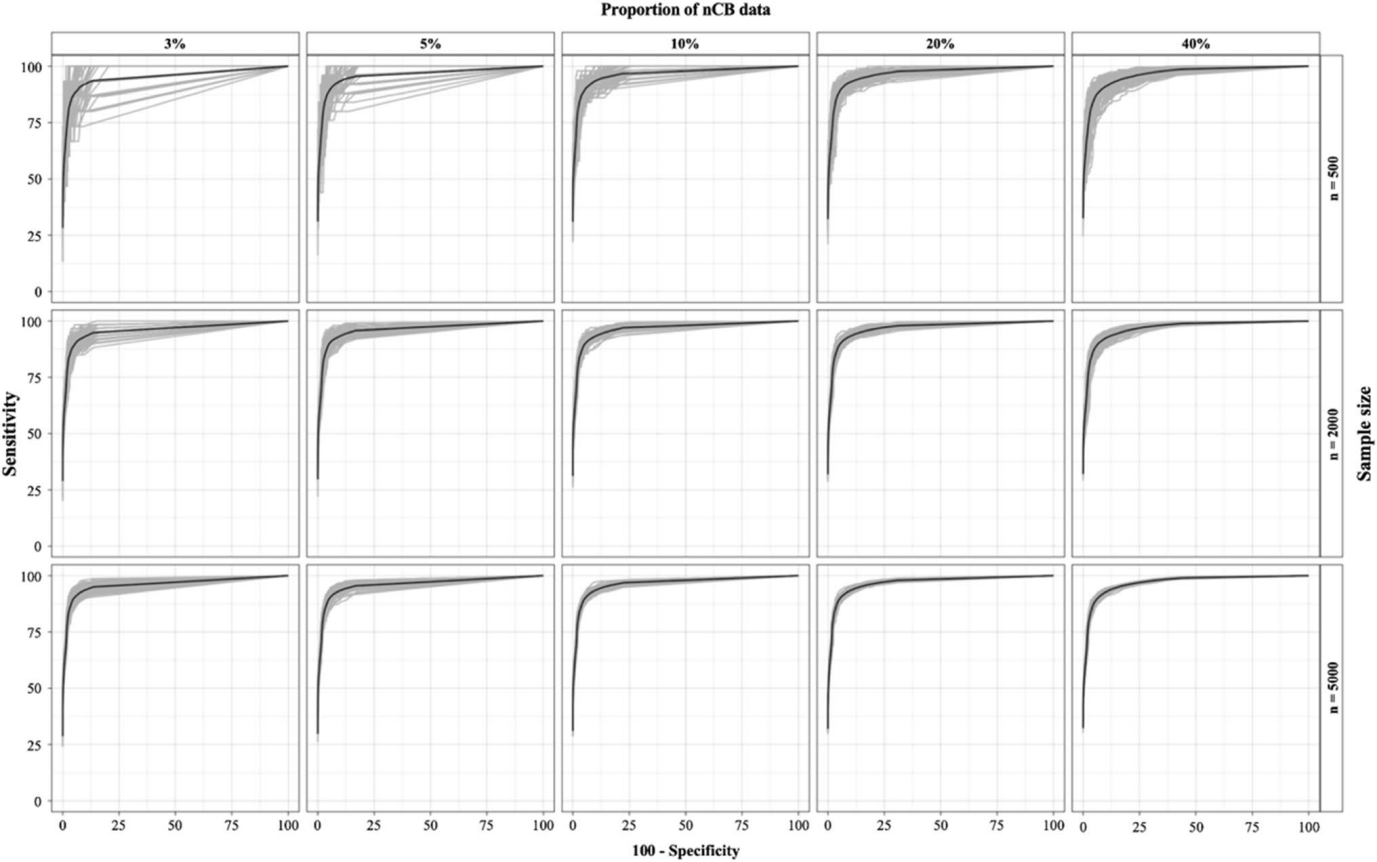
AUC estimates from conditions A1-C5. *Gray lines*: Individual AUCs. *Black line*: Representation of the mean AUC from the estimates of all AUCs

Figure 2 represents the distribution of 100 estimated AUCs for each condition (A1–C5). Table 4 shows the results of the ROC analysis for conditions A1 to C5 (means of 100 replications). The FMA produced remarkable AUC values in all conditions, ranging from .967 (condition A1) to .973 (condition C4). Specificity values ranged from .99 (condition A1) to .96 (condition C5). Sensitivity values were relatively high, ranging from .60 (condition A1) to .86 (condition C5). We compared the means of AUC, sensitivity, and specificity values by Welch's one-way ANOVA (Delacre et al., 2019). AUC values were equiprobable (*p* > 0.01) in all three sample sizes (*F* = 1.56; *p* = 0.21), as were sensitivity (*F* = 0.54; *p* = 0.57) and specificity (*F* = 2.7; *p* = 0.07). There was a clear positive association between sensitivity and prevalence of nCB data (*F* = 1367; *p* < .001), with mean differences ranging from 24.9 points for sensitivity (3% prevalence vs. 40% prevalence) to 2.5 points (3% vs. 5%). A significant negative association between specificity and prevalence was also observed (*F* = 1235; *p* < .001); however, in terms of units of measurement, the differences in specificity were small, with a maximum of 3.5 specificity points (3% prevalence vs. 40% prevalence) and a minimum of 0.25 points (3% vs. 5%).

**Table 4:** Results from ROC analysis (conditions A1–C5, Balanced scale)

Condition	Sample size	Proportion of nCB data	AUC (SD)	Sen	Spe	PPV	NPV	Valid data lost	nCB data cleaned
A1	500	0.03	0.967 (0.02)	0.60	0.99	0.70	0.99	0.8%	60.0%
A2		0.05	0.971 (0.01)	0.64	0.99	0.75	0.98	1.1%	63.8%
A3		0.1	0.972 (0.01)	0.69	0.99	0.85	0.97	1.4%	69.3%
A4		0.2	0.971 (0.01)	0.80	0.97	0.89	0.95	2.5%	80.2%
A5		0.4	0.966 (0.01)	0.84	0.96	0.93	0.90	4.5%	84.2%
B1	2,000	0.03	0.971 (0.01)	0.60	0.99	0.73	0.99	0.7%	60.1%
B2		0.05	0.971 (0.00)	0.62	0.99	0.77	0.98	1.0%	62.3%
B3		0.1	0.972 (0.00)	0.68	0.99	0.83	0.97	1.5%	68.1%
B4		0.2	0.972 (0.00)	0.81	0.98	0.89	0.95	2.5%	80.6%
B5		0.4	0.969 (0.00)	0.86	0.96	0.93	0.91	4.2%	85.5%
C1	5,000	0.03	0.972 (0.00)	0.60	0.99	0.73	0.99	0.7%	60.0%
C2		0.05	0.971 (0.00)	0.63	0.99	0.77	0.98	1.0%	62.5%
C3		0.1	0.972 (0.00)	0.68	0.99	0.84	0.97	1.4%	68.0%
C4		0.2	0.973 (0.00)	0.81	0.98	0.90	0.95	2.3%	80.5%
C5		0.4	0.970 (0.00)	0.86	0.96	0.93	0.91	4.2%	86.2%

**Note.** AUC = Area under the curve; Sen = sensitivity; Spe = specificity; PPV = Positive predictive value; NPV = Negative predictive value.

PPVs were reasonably high, ranging from 0.70 (A1) to 0.93 (A5). There was a consistent increase in PPV as nCB prevalence increased. On the other hand, NPVs were high, ranging from 0.90 (A5) to 0.99 (A1). A consistent decrease in NPV associated with increasing prevalence was also observed although the decrease was gradual in the 3% to 20% prevalence conditions (from 0.99 to 0.95), with a substantial jump at the 40% prevalence condition (0.90).

Table 4 shows two indicators, “valid data lost” and “nCB data cleaned,” to help interpret results. “Valid data lost” is the percentage of false positives and answers the question “in this condition, what percentage of valid cases have we erroneously discarded?” “nCBdata cleaned” is the percentage of true positives (i.e., sensitivity) and answers the question, “in this condition, what percentage of invalid cases have we managed to screen?” The proportion of valid data lost ranged from 0.7% (B1) to 4.5% (A5). At a prevalence of 3% to 20%, the proportion of valid cases lost was very low (2.5% in the worst case), while at a prevalence of 40%, it almost doubled (4.5% in the worst case). Finally, the proportion of correctly eliminated nCB cases ranged from 60% (A1) to 86% (C5).

#### Validity of the screener

**Fig. 3:**
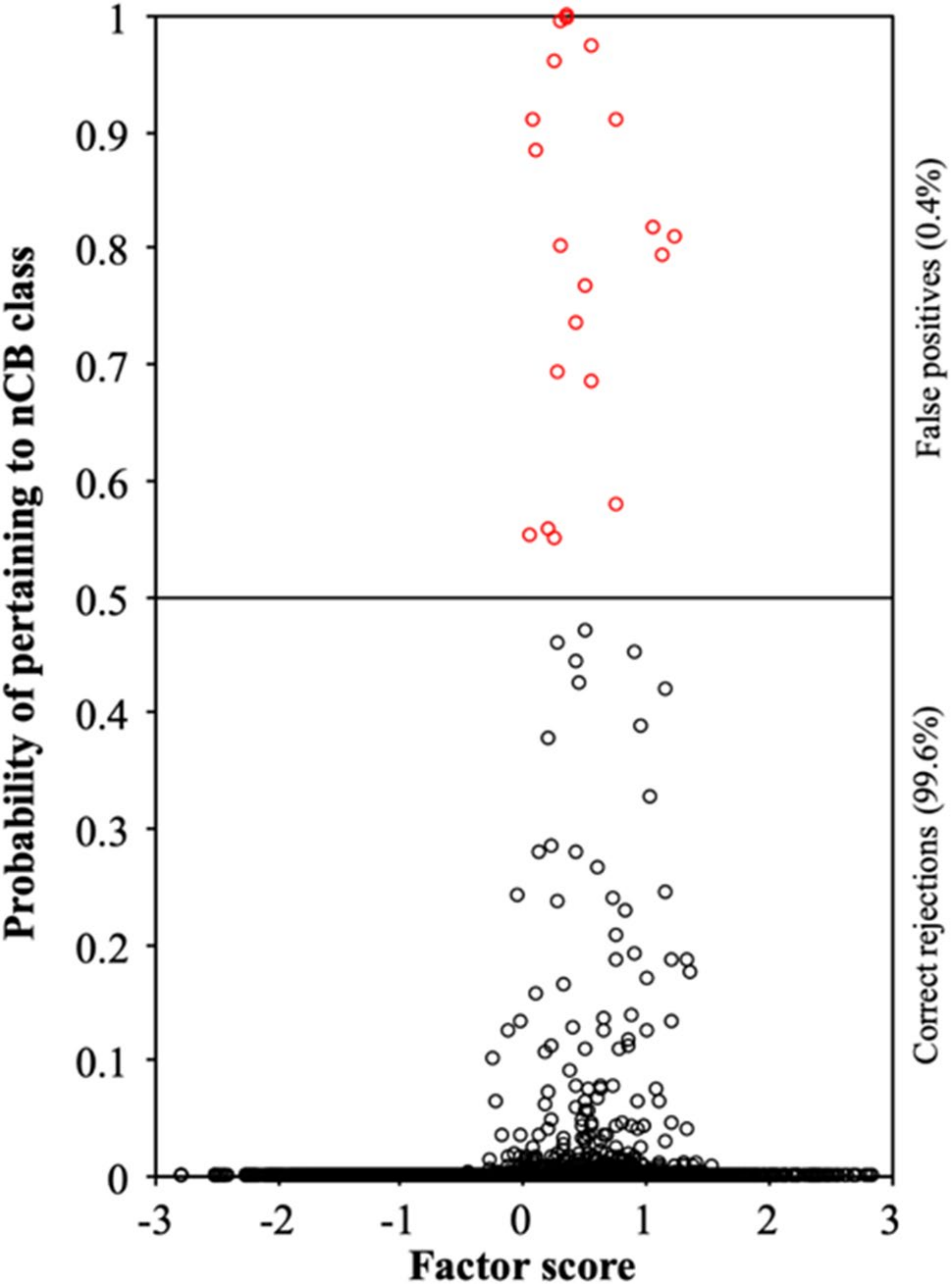
Scatter plot of factor scores and the probability of belonging to the nCB class, in the absence of nCB responses (mixed worded items; *n* = 5000)

First, we estimated FMA on the set of simulated data without nCB vectors to obtain a proxy for the null distribution of the screener. Thus, in this simulated sample, all cases are valid and the cases flagged by the FMA are always false positives. Next, we estimated the correlation between the screener (probability of belonging to an nCB class) and the factor scores of the one-dimensional model estimated on the full sample. Since the results were virtually the same for the three sample sizes, the results obtained with a sample size of 5000 are reported below. Figure 3 shows the scatterplot between the probability of belonging to a non-existent nCB class and the standardized factor scores, with false positives in red (first replication).

The mean correlation between the probability of belonging to the nCB class (FMA) and the factor scores was close to 0 (*r* = .06; ρ = .04), indicating that screener scores are not related to trait scores. In addition, the proportion of false positives was very low (0.4%). These results suggest that FMA produces very few classification errors when nCB data are not present.

#### Conditions D1–F5 (only positive items)

**Fig. 4:**
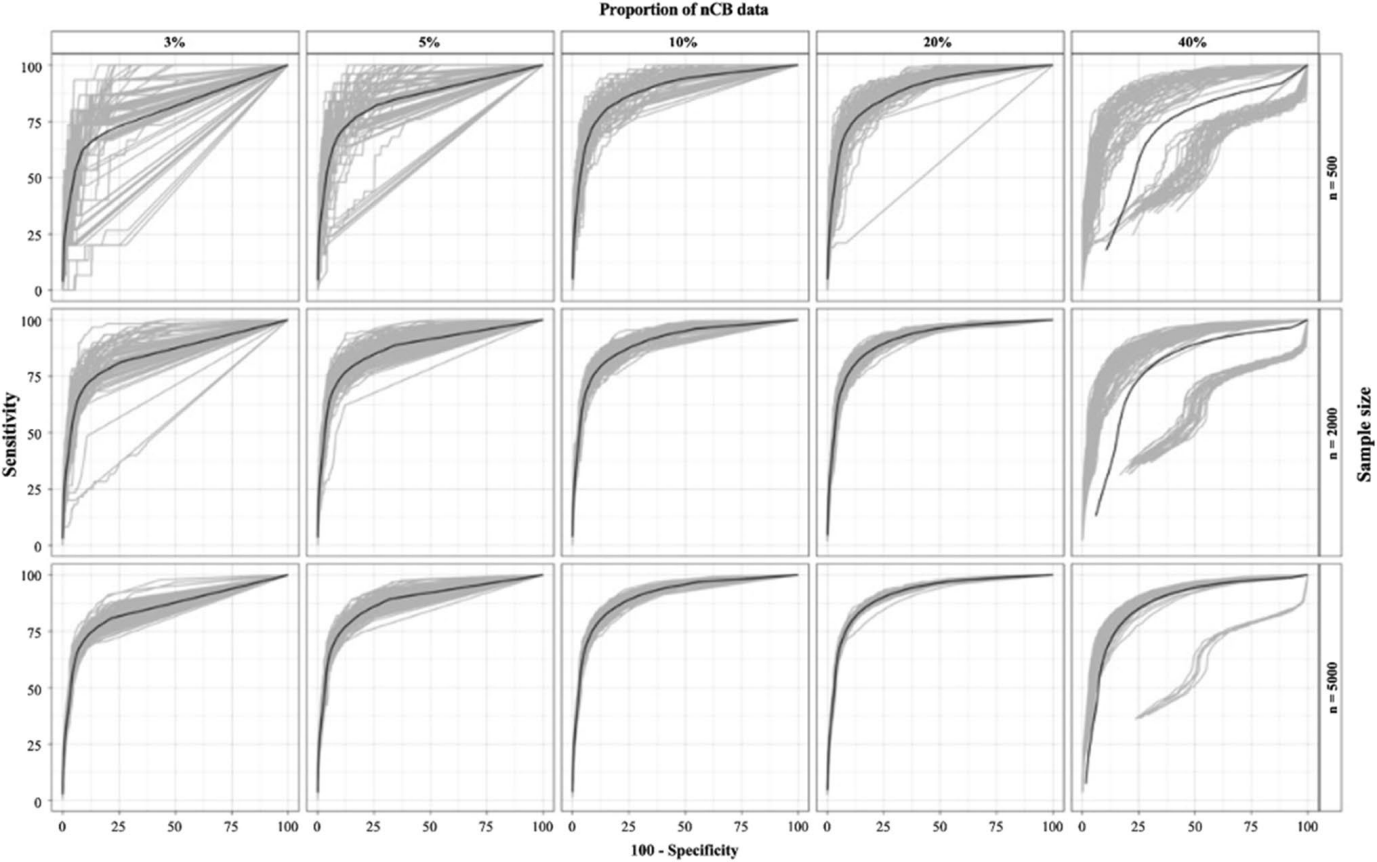
AUC estimates from conditions D1-F5. *Gray lines*: Individual AUCs. *Black line*: Representation of the mean AUC from the estimates of all AUCs

Figure 4 represents the distribution of the 100 estimated AUCs for each condition (D1–F5). Table 5 shows the results of the ROC analysis for conditions D1 to F5 (means of the 100 replications). FMA produced AUC values ranging from .916 (condition F4) to .736 (condition D5). Specificity values ranged from .67 (D5) to .98 (F1). Sensitivity values ranged from 0.40 (F1) to 0.74 (F5). In contrast to the other conditions, a significant proportion of the AUCs in the 40% prevalence condition had very low values. The result was a bimodal distribution of AUC values in the 40% prevalence conditions. This bimodality makes the estimates of the AUC means unreliable.

**Table 5:** Results from ROC analysis (conditions D1–F5, positive worded scale)

Condition	Sample size	Proportion of nCB data	AUC (SD)	Sen	Spe	PPV	NPV	Valid data lost	nCB data cleaned
D1	500	0.03	0.825 (0.11)	0.404	0.967	0.27	0.98	3.3%	40.4%
D2		0.05	0.875 (0.08)	0.504	0.961	0.40	0.97	3.9%	50.4%
D3		0.1	0.905 (0.02)	0.614	0.948	0.57	0.96	5.2%	61.4%
D4		0.2	0.897 (0.04)	0.674	0.929	0.70	0.92	7.1%	67.4%
D5		0.4	0.736 (0.16)	0.711	0.669	0.59	0.78	33.1%	71.1%
E1	2000	0.03	0.873 (0.05)	0.409	0.970	0.30	0.98	3.0%	40.9%
E2		0.05	0.897 (0.02)	0.471	0.970	0.45	0.97	3.0%	47.1%
E3		0.1	0.909 (0.01)	0.603	0.958	0.61	0.96	4.2%	60.3%
E4		0.2	0.913 (0.01)	0.702	0.933	0.72	0.93	6.7%	70.2%
E5		0.4	0.808 (0.01)	0.729	0.756	0.67	0.81	24.4%	72.9%
F1	5000	0.03	0.878 (0.02)	0.400	0.975	0.33	0.98	2.5%	40.0%
F2		0.05	0.899 (0.01)	0.461	0.971	0.46	0.97	2.9%	46.1%
F3		0.1	0.913 (0.01)	0.595	0.960	0.62	0.96	4.0%	59.5%
F4		0.2	0.916 (0.00)	0.703	0.938	0.74	0.93	6.2%	70.3%
F5		0.4	0.876 (0.08)	0.735	0.861	0.78	0.83	13.9%	73.5%

**Note.** AUC = Area under the curve; Sen = sensitivity; Spe = specificity; PPV = Positive predictive value; NPV = Negative predictive value.

When conditions with a prevalence of 40% were excluded from the analysis, a positive relationship between AUC and sample size were observed (*F* = 19.5; *p* < .001) although these differences were of small magnitude. There were a significant positive relationship between sensitivity and the prevalence of nCB cases (*F* = 632.2; *p* < .001), with mean absolute differences ranging from 7.5 sensitivity points (3% prevalence vs. 5% prevalence) to 28.8 points (3% vs. 20%). Differences in specificity were also significant and negative (*F* = 302.4; *p* < .001) but relatively small, ranging from 0.5 points in the 3% vs. 5% contrast to 3.9 points in the 3% vs. 20% contrast.

PPVs showed a strong positive relationship with prevalence, with a minimum of 0.27 (condition D1) and a maximum of 0.78 (condition F5). NPVs were stable across prevalence conditions, with a minimum of 0.92 (D4) and a maximum of 0.98 (D1). As expected from previous results, conditions with 40% prevalence showed substantially low NPVs (between 0.78 and 0.83). Finally, the proportion of valid data lost ranged from 2.5% (F1) to 33% (D5). The proportion of cleaned data ranged from 40% (F1) to 73.5% (F5).

#### Validity of the screener

**Fig. 5:**
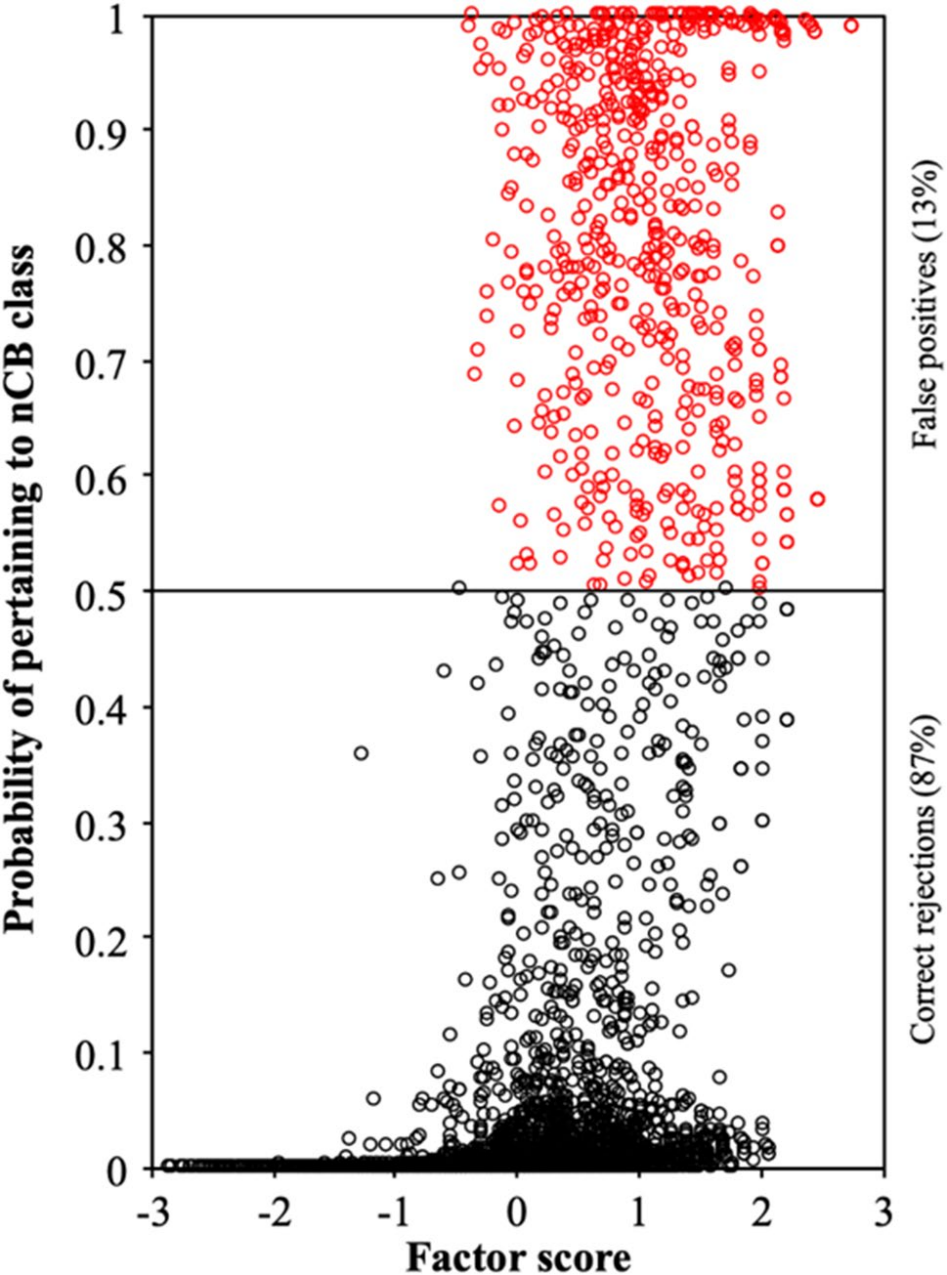
**S**catter plot of factor scores and the probability of belonging to the nCB class, in the absence of nCB responses (positive worded items; *n* = 5000).

Figure 5 shows screener scores plotted against factor scores in a sample without nCB cases (first replication, *n* = 5000). A substantial number of false positives (13%) were found, clustered between 0 and +2 SDs from the factor mean. The correlation between the screener and the factor scores was .51 (ρ = .39). These results suggest that FMA, on scales without reverse-keyed items, misclassifies individuals with moderate to high scores on a trait, possibly confusing these scores with acquiescent response patterns.

## Study 2 (real data)

We devoted this study to testing the performance of FMA as a screener of nCB responses using real data from 5928 individuals assessed on a theoretically unidimensional construct (generalized optimism). To this end, we analyzed the structure and properties of the data before and after screening based on the following assumptions:

1. nCB responses contribute to the deflation of correlations between items measuring the same construct. Random styles produce an overall deflation as large as the proportion of random respondents in the sample. However, the most pronounced deflation would arise from (dis)acquiescent styles, as these vectors yield unexpected subsets of positive correlations between regular and reverse-keyed items (Arias et al., 2020a).
2. nCB responses are not related to the meaning of items and, consequently, will produce response vectors that the theoretical model finds to be highly unexpected, which has two consequences: (a) a substantial deterioration of the one-dimensional model fit and (b) the need to specify additional factors to account for the unexpected responses (Reise et al., 2016).

Given these assumptions, if the FMA proves useful as a screening tool, we expect that removing the nCB cases will lead to:

1. a substantial increase in the fit of the one-dimensional model.
2. a substantial increase in the estimates of convergence between positive and reverse-keyed items.
3. a substantial reduction in the variance captured by additional factors (e.g., specific factors in bifactor models) and a considerable increase in the reliable variance of the substantive factor.

### Participants and instruments

We used data from the first wave of the Longitudinal Internet Studies in the Social Sciences panel of CentERdata at the University of Tilburg. The raw data are available upon request at www.lissdata.nl. The first sample consisted of 5928 participants with no missing data (53.4% women) aged 16 to 92 (M = 49.6; SD = 17.4).

Participants completed the Dutch version of the Life Orientation Test-Revised (LOT-R; Scheier et al., 1994). LOT-R contains six items (half reverse-keyed) assessing generalized outcome expectations (e.g., “In uncertain times, I usually expect the best”). Respondents must indicate their agreement with each statement on a five-point scale (from “strongly disagree” to “strongly agree”). Although the theoretical structure of optimism is unidimensional (Scheier et al., 1994), some studies (e.g., Creed et al., 2002) have proposed a multidimensional structure in which optimism and pessimism are separable dimensions.

### Data analysis

**Fig. 6:**
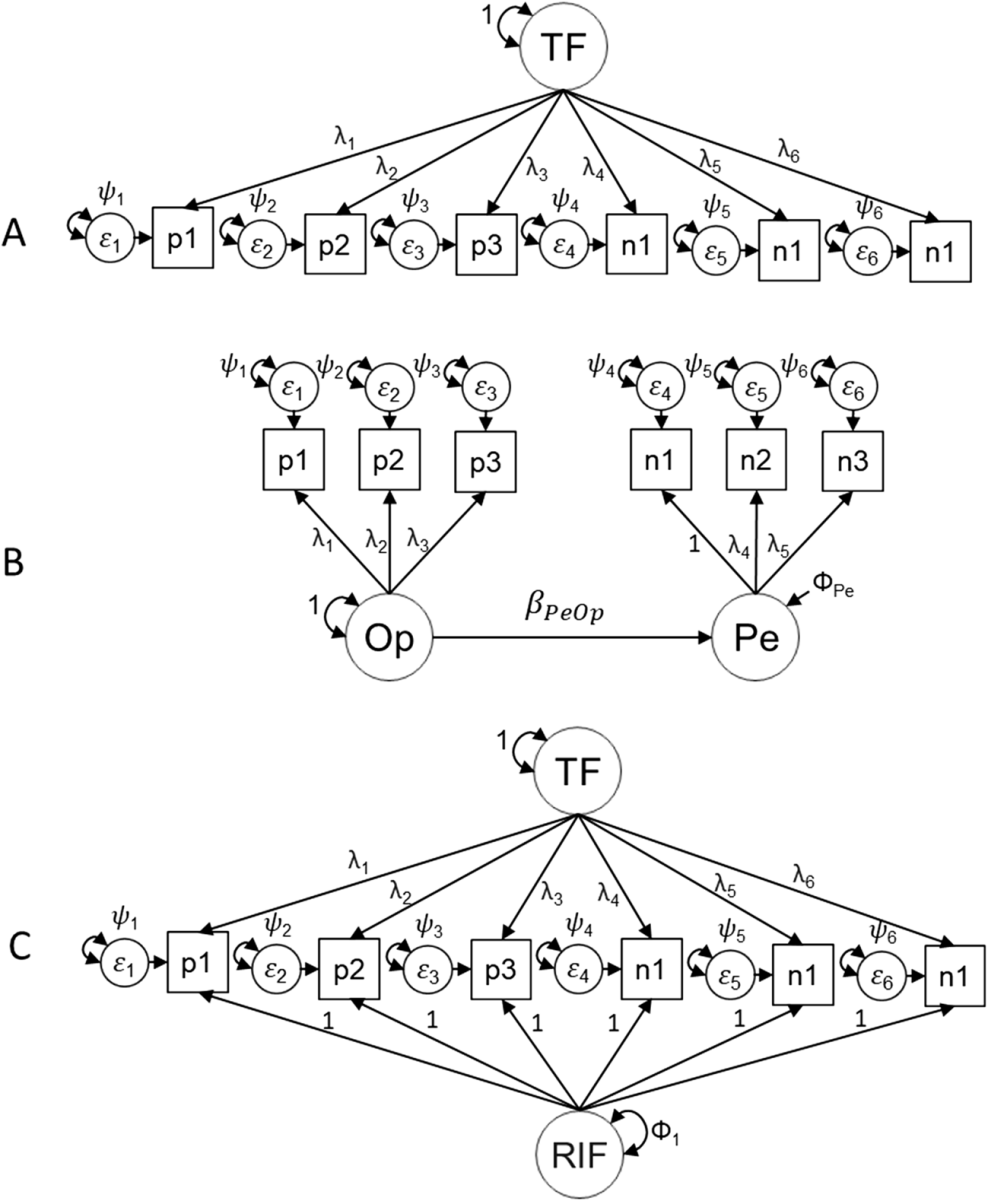
Conceptual representations of confirmatory factor models. TF, trait factor; p1–p3, positive worded items; n1–n3, reverse-keyed items; Op, optimism; Pe, pessimism; RIF, random intercept factor

We first estimated three factor structures (Fig. 6). Panel A in Fig. 6 represents the unidimensional model, which is consistent with the theoretical structure of the construct. Panel B represents the restricted correlated trait correlation methods minus 1 model (restricted CT-C(M-1); Eid, 2000; Geiser et al., 2008). CT-C(M-1) is a structural equation model with two factors, one measured using positive items (optimism) and the other using reverse items (pessimism), with optimism acting as a predictor of pessimism.

In the CT-C(M-1) model, latent regression path (*β*) is an estimator of the convergence between estimates of the same trait (optimism–pessimism) measured using different methods (positive and reverse-keyed items). Consequently, the residual variance of pessimism represents the common variance associated with the measurement method as opposed to the common variance explained by the trait represented by *β*. An excess of method variance will produce a substantial deflation in the relationship between factors because trait and method factors are uncorrelated.

Finally, Panel C illustrates a random intercept factor analysis (RI-FA) model (Maydeu-Olivares & Coffman, 2006). RI-FA is a model with two orthogonal general factors, where all items load on both a general factor representing the substantive trait and a RI factor whose loadings are set to 1 (reverse-keyed items are not recoded). The RI factor attempts to model systematic individual differences in response scale usage by decomposing the intercept into two components: a component that is constant across individuals but varies across items (*μ*_*i*_) and a component that is constant across items but varies across individuals (*ξ*_*j*_). *ξ*_*j*_ can be interpreted as individual differences in the use of response scale and are independent of the trait factor (Maydeu-Olivares & Coffman, 2006). nCB response styles are consistent with this definition, as they are response patterns that act homogeneously across items although their intensity may vary between individuals. All factor models were estimated with MPlus 8.2 (Muthén & Muthén, 1998–2017) using weighted least square mean and variance adjusted (WLSMV).

To assess the RI-FA results, in addition to fit, we estimated the explained common variance (ECV) of both factors and the hierarchical omega (*ω*_*H*_). ECV is an estimate of the proportion of common variance captured by the trait factor. A minimum ECV of 0.80 has been recommended for data to be considered essentially unidimensional (Rodriguez et al., 2015). *ω*_*H*_ estimates the proportion of reliable variance that can be attributed to the general factor: *ω*_*H*_ greater than 0.70 is necessary to ensure that scores on the general factor are reliable estimators of the substantive trait (Reise et al., 2013).

The models described were estimated using two databases: one containing the original, unscreened data (which we will call “raw data”) and the other containing only the cases that were not flagged as nCB by FMA (“clean data”).

### Results

After examining the sources of local misfit in the unidimensional model, we released the correlation between the residuals of two items that showed clear similarities in their wording in addition to a modification index and a standardized expected parameter of change greater than 10 and 0.3, respectively (Saris et al., 2009). Table 6 presents the fit of three models estimated from raw data. In the raw sample, the one-dimensional model showed the poorest fit (RMSEA = 0.16 and CFI = 0.91). The restricted CT-C(M-1) model showed a significantly superior fit (RMSEA = 0.06 and CFI = 0.99). The standardized regression path was – 0.62. This value is very low considering that optimism and pessimism are theoretically opposite poles of the same construct. Finally, the RI-FA model showed the best fit (RMSEA = 0.04 and CFI = 0.99), with 80% of the common variance explained by the trait factor and 20% explained by idiosyncrasies in the use of the response scale. *ω*_*H*_ was poor (0.65).

**Table 6:** Results from factor analysis

Sample	Model	fp	CFI	TLI	RMSEA	SRMR	*b* (*R*^*2*^)	ECV	ω_H_
Raw data (*n* = 5928)	CFA 1-Factor	31	0.912	0.834	0.162	0.046			
	R-CTCM-1	32	0.990	0.978	0.059	0.015	0.62 (0.38)		
	RI-FA	32	0.994	0.987	0.045	0.011		0.800	0.650
Clean data (*n* = 5541)	CFA 1-Factor	31	0.988	0.977	0.063	0.017			
	R-CTCM-1	32	0.994	0.987	0.048	0.012	0.90 (0.81)		
	RI-FA	32	0.995	0.989	0.045	0.011		0.940	0.760

**Note.** fp = free parameters; CFI = Comparative Fit Index; TLI = Tucker-Lewis Index; RMSEA = Root Mean Squared Error of Approximation; SRMR = Standardized Root Mean Squared Residual; *b* = Regression path; *R*^*2*^ = R-squared ; ECV = Explained Common Variance; ω_H_ = Hierarchical omega.

Taken together, these results suggest that the unidimensional model should be rejected in favor of one of the multidimensional models. The RI-FA model suggests a single substantive factor that explains 80% of the common variance, but with too low reliability and a non-ignorable amount of spurious systematic variance (20%). The CT-C(M-1) model revealed a significantly lower-than-expected relationship between optimism and pessimism. This low convergence suggests either an excessively large method effect or the possibility that optimism and pessimism function as separate dimensions.

Next, we estimated FMA on the raw data, classifying 6.5% of the cases (*n* = 387) as nCB respondents. Table 6 shows the results of the factor analyses on the screened sample (*n* = 5541). The one-dimensional model showed a reasonably good fit (RMSEA = 0.06 and CFI = 0.98), very close to the fit of the CTCM-1 (RMSEA = 0.04 and CFI = 0.99) and RI-FA (RMSEA = 0.04 and CFI = 0.99) models. For the RI-FA model, the ECV of the trait factor increased to 0.94 and *ω*_*H*_ improved to 0.76. This result implies a reduction in the variance of the RI factor from 20% to 6%, and sufficient reliability for a proper interpretation of the trait factor scores. In the CTC-(M-1) model, the standardized regression path value increased from 0.64 to 0.90.

Considering these results, after screening, it is not clear that the hypothesis of one-dimensionality can be rejected since the fit of the unidimensional model is now very close to that of the multidimensional models. Moreover, the high convergence between the two forms of the test does not allow us to ensure their empirical separability. On the other hand, after removing 6.5% of the cases, most of the prediction residual in the CTC-(M-1) model and the variance of the RI factor in the RI-FA model disappeared. These results have two implications: First, most of the prediction residuals in the CTC-(M-1) model are not due to the use of reverse-keyed items but are, instead, related to the highly inconsistent responses of a small group of individuals detected by FMA. Second, most of the systematic variance captured by RI-FA does not represent a phenomenon generalizable to the entire sample but is rather generated by the highly anomalous responses of a minority subgroup.

### Discussion

The discussion will be presented as follows: First, we will discuss the main findings of each study separately. Next, we will address the strengths and limitations of FMA as a method for detecting nCB responses. Finally, we will evaluate the limitations of the study and possible directions for future research.

## Discussion of Study 1

Using data from mixed-worded items (positive and reverse-keyed items), the restricted FMA model showed reasonably satisfactory sensitivity and excellent specificity in all prevalence conditions. Furthermore, these results suggest that the performance of the model is stable across different sample sizes. In addition, the model proved to be robust, as it converged without problems even in the absence of nCB data and had a minimal proportion of false positives. Scores on the screener were independent of scores on the latent trait – a necessary condition for avoiding selection bias – and the false-positive rate was very low (0.4%).

In contrast, the results for positively worded scales were poor. The specificity values were high, but not satisfactory given the importance of avoiding erroneous screening of valid cases. On the other hand, the model performed significantly poorly at an extreme prevalence of nCB data (40%), where it produced an unacceptable false positive rate. This conclusion is reinforced by the correlation between the screener and the trait (.51), which carries a clear risk of selection bias, especially in individuals with medium to high levels of the trait. In the absence of reverse-keyed items, this result is to be expected because it is very difficult or impossible to distinguish nCB dis-acquiescent respondents from individuals with very high or low levels of the trait (Reise et al., 2016). In addition to the differences in the magnitude of the intercepts, the differences in the signs of the factor loadings of positive and reverse items are the main source of information with which the model classifies responses. The better performance of the FMA on balanced scales is due in part to the fact that inconsistent responses produce a pattern of factor loadings with different signs than those expected on balanced scales. Thus, the equality of loadings as a source of information for classification is lost on scales with items phrased with the same semantic polarity. Therefore, we do not recommend using FMA in scales without reverse-keyed items.

## Discussion of Study 2

In this study, we compared the factor structure of the data before and after screening the nCB cases. The FMA flagged 6.5% of the sample as nCB respondents. Although this percentage is relatively small, it caused a very strong bias in the results of the analysis. After removing the nCB data, the unidimensional model went from a very poor fit to a reasonably good fit, and the spurious systematic variance found in the CT-(M-1) and RI-FA models was substantially decreased.

These results suggest that FMA was able to detect the response vectors that contributed most to data bias. Moreover, the flagged cases accounted for most of the spurious systematic variance in the CT-C(M-1) and RI-FA models: once the nCB data were removed, the data became sufficiently consistent to not reject the unidimensional hypothesis. In conclusion, the screening of the data allowed the removal of most of the systematic error variance that was confounding the analyses on the full sample.

### Strengths and limitations of FMA

The FMA model presented here has demonstrated several strengths in the detection of nCB responses. In the simulated data, FMA showed reasonably high diagnostic accuracy across different prevalence of nCB cases. Although there is a positive relationship between the sensitivity of the model and the prevalence of nCB data, the specificity levels were consistently very high. This result suggests that the higher the prevalence of nCB cases, the more sensitive the model is while maintaining a very low false positive rate. In real data, FMA was able to detect a minority of individuals with highly aberrant responses. Although further research is needed to determine the exact limits and potential of FMA for the detection of nCB responses, our results suggest that it is a promising method.

Another strength of FMA is its foundation on explicit theoretical assumptions that can be tested empirically. In this study, we have presented a restricted specification of FMA that attempts to model the processes underlying nCB response styles by determining the relationship between item meaning and the parameters of the measurement model. This is not an inflexible or closed specification; rather, it is open to modification and improvement by other researchers, who could make changes based on different theoretical premises. Additionally, the FMA model is flexible enough to be adapted to other types of problematic responses, such as those due to reading and processing difficulties of reverse-keyed items as demonstrated by Steinmann et al. (2021) or combined with other models to account for different types of nCB data, such as errors due to inattention and difficulty in item verification (Baumgartner et al., 2018). Finally, because FMA is a restricted model with a fixed specification of classes and factors (only the number of items changes), it is relatively easy to estimate the model using well-known software such as MPlus (see Appendix A for an annotated example of the model’s syntax).

The main limitation of FMA is that it can work well only when there are reverse-keyed items on a scale. In the case of an instrument with only positive items, FMA will find it difficult to distinguish, based on the data alone, an acquiescent response vector from a thoughtful response that truly denotes a very high or very low level of the trait. Other screeners also have difficulty assessing the validity of repetitive responses in the absence of reverse items (Conijn et al., 2019). One solution to this problem might be to include reverse-keyed items in all assessments to aid in the detection of inconsistent responses, as has been traditionally recommended (e.g., Baumgartner & Steenkamp, 2001; Cronbach, 1946; Messick, 1991). In addition, these reverse-keyed items could be used only as a mechanism for controlling response styles without the need to include them in the final scores if the researcher or practitioner so chooses.

Finally, for FMA to work properly, the overall factor model must be correctly specified. The hypothesis of unidimensionality represented by the overall model in FMA must be essentially true in order to prevent the nCB class from capturing response vectors that are valid but inconsistent with a misspecified overall model (e.g., due to the existence of unmodeled substantive multidimensionality). To this end, prior to estimating FMA, it is necessary to perform a thorough analysis of the dimensionality and structure of the data to (a) ensure that the proportion of common variance captured by the trait factor after partialization of the residual non-substantive variance is sufficient to maintain the essential unidimensionality hypothesis (this can be done using a RI-FA model such as the one used in Study 2) and (b) identify and incorporate substantive sources of local misfit into the overall model (e.g., high residual correlations due to narrow facets beyond the general trait factor or semantic similarity between items).

## Study limitations and directions for future research

One limitation of the present study is that our FMA model uses intercepts to model ordinal data. Although the estimators for continuous data perform similarly to their analogs for ordinal variables when there are five or more response categories (DiStefano, 2002; Johnson & Creech, 1983; Zumbo & Zimmerman, 1993), it is necessary to investigate the performance of FMA on items with four or fewer categories. It is possible to adapt FMA to ordinal variables by using thresholds instead of intercepts (e.g., by making the threshold pattern the same for all items). There are promising mixture models suitable for use with ordinal variables. For example, Ulitzsch et al. (2022) have developed an item response theory mixture model suitable for detecting careless responses to categorical items.

Moreover, our FMA model assumes that a response style manifests itself across all items in a test. However, there may be degrees of nCB response severity, such that an individual may exhibit a response style only in a portion of the test (e.g., due to fatigue in long test sessions; Hong et al., 2020). For long test sessions, one possible solution is to estimate an FMA model for each unidimensional scale and then compare classifications, thus ranking cases as a function of the prevalence of nCB responses across different item sets.

Furthermore, we investigated the performance of FMA under different prevalence levels of nCB data and response styles. However, it is necessary to increase the variety of possible conditions to clearly understand the limits of the model, especially in cases in which the data are highly asymmetric, such as clinical assessments done on the general population. In addition, the residual variances of the items are assumed to be homoscedastic across classes to avoid empirical underidentification problems in cases where the nCB class size is small relative to the number of parameters in the mixture model. However, it is necessary to further investigate the role of residual variances in detecting nCB responses, as well as the conditions under which it is safe to relax the model constraints.

Finally, perhaps the most important limitation of this study is that we lack unequivocal evidence that FMA truly captures nCB responses. This problem is common to all studies involving nCB data detection. The evidence from real data suggests that FMA correctly detects highly unusual response patterns; however, we cannot conclude with certainty that these response patterns are caused by inattention or carelessness. A worthy research goal, both for FMA and other screeners, is to obtain unambiguous evidence of their classification validity. Although achieving this goal is extremely difficult because research in this area often requires large sample sizes, relevant insights can be obtained through laboratory studies or individual interview-based studies that do an in-depth analysis of the processes underlying item responses (Arias et al., 2020b; Baumgartner et al., 2018; Curran and Hauser, 2019).

- The data and materials for study 1 are available at
- https://osf.io/fy59v/?view_only=580d587f6c1e4b49beae6d270ee07078
- The data and materials for study 2 are available at
- www.lissdata.nl (upon request)
- None of the studies was preregistered

### Supplementary information

ESM 1 (PDF 11 kb)
