# Supplementary material for: Detecting non-content-based response styles in survey data: An application of mixture factor analysis
Source: Behav Res Methods. 2023 Dec 21;56(4):3242–58. doi: 10.3758/s13428-023-02308-w (PMC11133220; doi:10.3758/s13428-023-02308-w)
Supplement: Supplementary file 1 — (PDF 11 kb) [file 13428_2023_2308_MOESM1_ESM.pdf]

Appendix A

This is an annotated example of MPlus code for estimating the FMA on a set of 14 items.

```
VARIABLE:
NAMES ARE P1 P2 P3 P4 P5 P6 P7 N1 N2 N3 N4 N5 N6 N7; !In this example,
P=positive item, N=reverse-keyed item. IMPORTANT: do not recode reverse-
keyed items before analysis.
USEVARIABLES ARE P1 P2 P3 P4 P5 P6 P7 N1 N2 N3 N4 N5 N6 N7;
CLASSES = c(2);

MODEL:

%Overall%
FACTOR BY P1-N7; !This is the general one-dimensional model
!If any correlated residual, specify it here

%c#1% !This class groups respondents who have used non-content based response
styles.
FACTOR BY
P1* P2* P3* P4* P5* P6* P7* N1* N2* N3* N4* N5* N6* N7* (L1); !All factor
loadings have the same value
FACTOR@1; !Factor variance set to 1
[P1-N7] (T1); !All intercepts have the same value
[FACTOR@0]; !Factor mean set to 0

%c#2% !This class groups thoughtful respondents.
FACTOR BY
P1* P2* P3* P4* P5* P6* P7* N1* N2* N3* N4* N5* N6* N7*; !All factor loadings
are freely estimated
FACTOR@1; !Factor variance set to 1
[P1-N7]; !All intercepts are freely estimated
[FACTOR@0]; !Factor mean set to 0

ANALYSIS:
TYPE = MIXTURE;
STARTS = 800 800; !If the best loglikelihood is not replicated (see the MPlus
output), increase the starts
ESTIMATOR IS MLR;
STITERATIONS = 200;
PROCESS = 8 (STARTS);

OUTPUT: STANDARDIZED; TECH1;
SAVEDATA: FILE IS nCBprobs.XLS; !File with the results of the classification
(excel format)
SAVE IS cprob; !Mplus saves the a posteriori probabilities of belonging to class 1
and 2, for each case.
FORMAT IS free;
```
